# Supplementary material for: Effects of DNA Topology on Transcription from rRNA Promoters in Bacillus subtilis
Source: Microorganisms. 2021 Jan 1;9(1):87. doi: 10.3390/microorganisms9010087 (PMC7824091; doi:10.3390/microorganisms9010087)
Supplement: Supplementary file 1 [file microorganisms-09-00087-s001.pdf]

microorganisms

MDPI

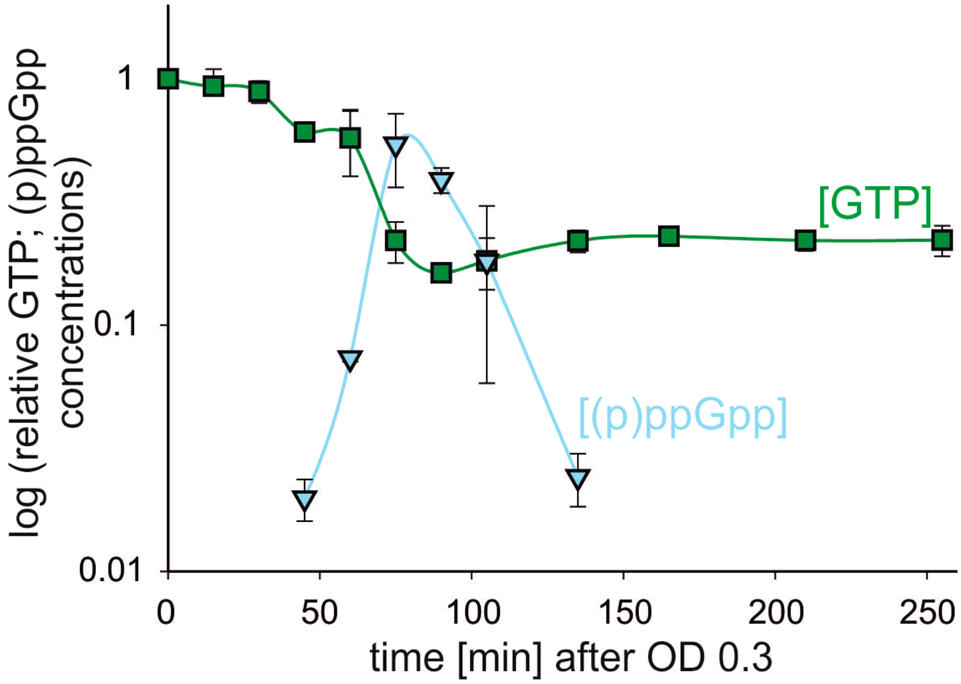

Figure S1. Relative GTP and (p)ppGpp levels after entry into the stationary phase.

Relative GTP concentration (green squares) and relative (p)ppGpp concentration (blue triangles) in wt genetic background after entry into stationary phase. The GTP concentration is normalized to 1 at time 0. The GTP concentration data are from two independent experiments; the graph shows the mean and the error bars the range. (p)ppGpp concentration are from the same experiments as GTP (Figure 1), shown relative to GTP.

Microorganisms **2020**, *8*, 87; doi:10.3390/microorganisms9010087 www.mdpi.com/journal/microorganisms

microorganisms

MDPI

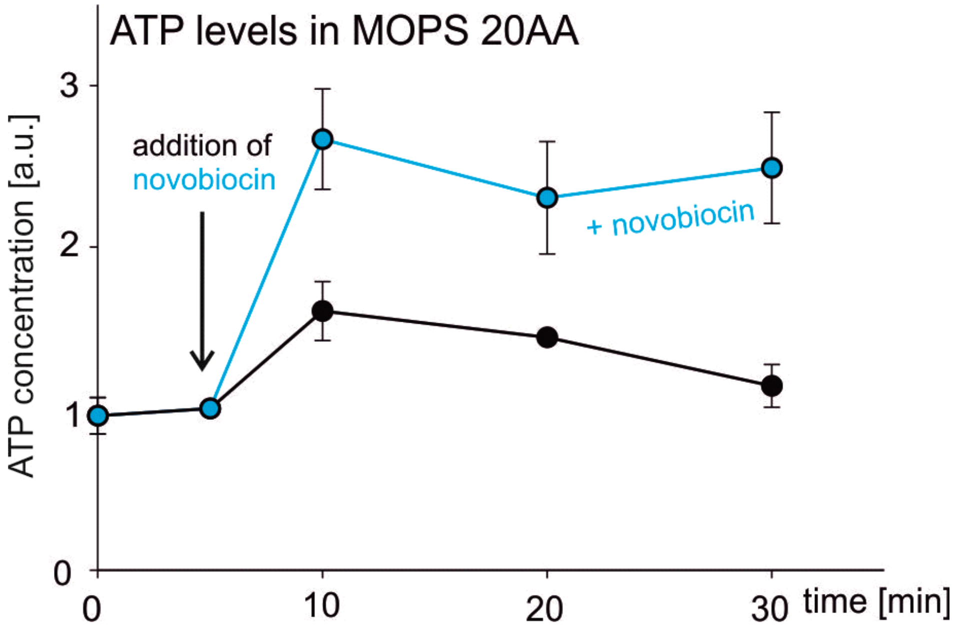

Figure S2. Effect of novobiocin-induced relaxation of chromosome on ATP levels.

Cells were grown in MOPS media supplemented with 20 amino acids with [^{32}P] H_3PO_4 to early exponential phase (OD_{600} ~ 0.3). At time 5 min the cells were treated with novobiocin (5 µg/ml). Levels of ATP were determined by TLC from the same TLC plates as GTP levels in Figure 2D. The ATP level at time 5 min was set as 1. Results are averages from two measurements. The error bars show the range.

microorganisms

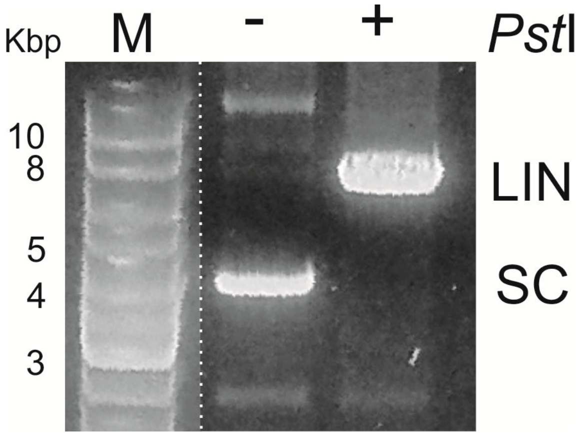

Figure S3. SC and LIN promoter DNA on agarose gel.

100 ng of DNA was resolved on 0.8 % agarose gel. M, DNA Mw marker (GeneRuler DNA Ladder Mix), -/+ the treatment with restriction enzyme *Pst*I. The DNA gel was stained with GelRed. The marker was assembled electronically with the rest of the gel – indicated with the dotted line.

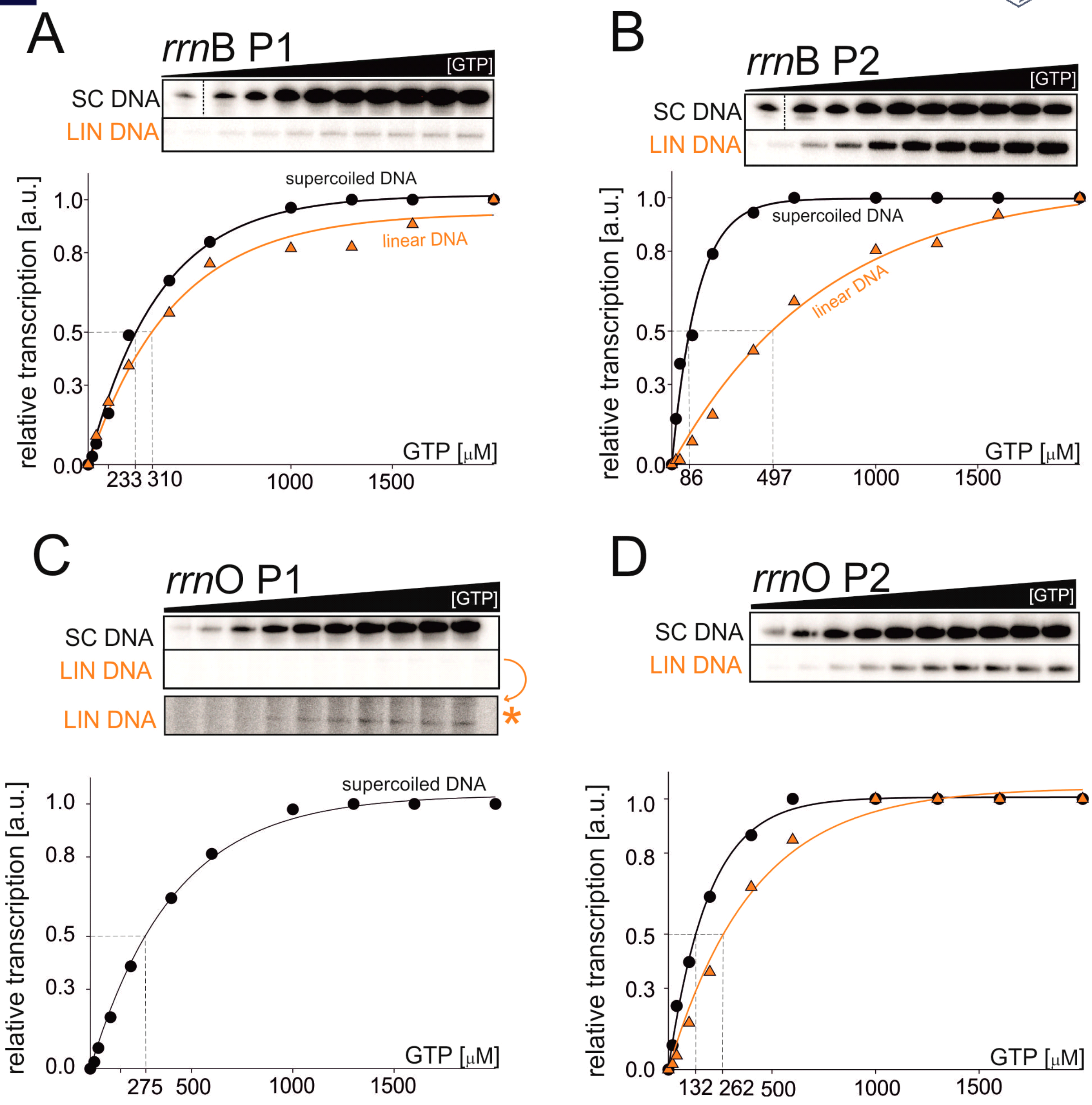

Figure S4. The affinity of RNAP for iNTP *in vitro* changes on different DNA templates.

Multiple-round transcriptions as a function of GTP concentration: representative primary data and their graphical comparison for *rrn*B P1 **(A),** *rrn*B P2 **(B),** *rrn*O P1 **(C)** and *rrn*O P2 **(D)**. For LIN *rrn*O P1 levels of transcripts were close to the background so that they were almost not detectable. The panel marked with the asterisk (*) shows the same data as the panel above but adjusted for brightness for better visibility. The K_{GTP} values are in Supplementary Table 1. The experiment was performed in at least three biological replicates.

Table S1: The K_{GTP} values for the promoters tested in the transcriptions *in vitro*.

The values were calculated at least from three independent experiments, showing the mean and ± SD. ND means "not detectable", as primary signal from transcription from LIN variant of *rrn*O P1 was too close to background (see Figure S4C).

**K_{GTP} [μM]**	**SC ± SD**	**LIN ± SD**	**Ratio SC/LIN**
P*veg*	36 ± 9	511 ± 78	~ 14x
*rrn*B P core	277 ± 24	440 ± 25	~ 1.5x
*rrn*B P1	242 ± 31	361 ± 46	~ 1.5x
*rrn*B P2	62 ± 13	427 ± 61	~ 7x
*rrn*O P1	240 ± 18	ND	
*rrn*O P2	98 ± 22	269 ± 8	~ 3x

Microorganisms **2020**, *8*, 87; doi:10.3390/microorganisms9010087 www.mdpi.com/journal/microorganisms

Table S2. Alternative σ factor-dependent promoters used in the study.

Promoter consensus elements are in bold. The 3′ ends of the sequences are transcription +1 positions.

σ factor	promoter	Product function*	Sequence of promoter	Reference
B	P*trxA*	Protection of proteins against oxidative stress	TCAG**GTTTTA**AAACAGCTCCGGCA**GGGCAT**GGTAAAGTAC**A**	[1]
D	P*motA*	Motility and chemotaxis	AATGTCCC**TAAA**GTTCCGGGCACCAAA**ACCGATAT**TAACCAT**A**	[2]
E	P*spoIIID*	Regulator of mother cell expression	ATATTCCCAAAAGAATGCTA**ATACAC**TGTTAC**A**	[3]
F	P*spoIIQ*	Forespore encasement by the spore coat	TT**GTATAT**ATTTTCAGAAAAGTGTT**CAGAATGT**TGCT**G**	[4]
H	P*spoVG*	Cell division, control of sporulation initiation	AAAAACGAGC**AGGATTT**CAGAAAAAATCGTG**GAAT**TGATACACT**A**	[5]
N	P*zpaB*	DNA gyrase#	**ATTTACGTTT**TAGAAAGACTA**GATATAA**AGATTAC**G**	[6]
N	P*zpbY*	unknown#	**ATTTACGTTT**TCAAAGGCACA**GATATAA**TAAC**A**	[6]
N	P*zpdG*	DNA pol III#	**ATTTACGTTT**TTGCCGGTCCA**GATATAA**ATACTTT**G**	[6]
N	*sigN* P3	Sigma factor#	TTTTCG**TTTACGTTT**CTATTTCTCTA**GATAAAA**TCATTAA**G**	[6]

*according to [7].

as published in [6].

Supplementary references:

1. Scharf, C.; Riethdorf, S.; Ernst, H.; Engelmann, S.; Völker, U.; Hecker, M. Thioredoxin is an essential protein induced by multiple stresses in Bacillus subtilis. *J. Bacteriol.* **1998**, *180*, 1869–1877, doi:10.1128/jb.180.7.1869-1877.1998.

2. Gilman, M.Z.; Wiggs, J.L.; Chamberlin, M.J. Nucleotide sequences of two bacillus subtilis promoters used by bacillus subtilis sigma-28 RNA polymerase. *Nucleic Acids Res.* **1981**, *9*, 5991–6000, doi:10.1093/nar/9.22.5991.

3. Kunkel, B.; Kroos, L.; Poth, H.; Youngman, P.; Losick, R. Temporal and spatial control of the mother-cell regulatory gene spoIIID of Bacillus subtilis. *Genes Dev.* **1989**, *3*, 1735–1744, doi:10.1101/gad.3.11.1735.

4. Amaya, E.; Khvorova, A.; Piggot, P.J. Analysis of Promoter Recognition In Vivo Directed by F of Bacillus subtilis by Using Random-Sequence Oligonucleotides. *J. Bacteriol.* **2001**, *183*, 3623–3630, doi:10.1128/JB.183.12.3623-3630.2001.

5. Haldenwang, W.G.; Losick, R. Novel RNA polymerase σ factor from Bacillus subtilis. *Proc. Natl. Acad. Sci. U. S. A.* **1980**, *77*, 7000–7004, doi:10.1073/pnas.77.12.7000.

6. Burton, A.T.; DeLoughery, A.; Li, G.W.; Kearns, D.B. Transcriptional regulation and mechanism of sigN (ZpdN), a pBS32-encoded sigma factor in bacillus subtilis. *MBio* **2019**, *10*, e01899-19, doi:10.1128/mBio.01899-19.

7. Zhu, B.; Stülke, J. SubtiWiki in 2018: From genes and proteins to functional network annotation of the model organism Bacillus subtilis. *Nucleic Acids Res.* **2018**, *46*, D743–D748, doi:10.1093/nar/gkx908.
